# Interindividual differences in incentive sensitivity moderate motivational effects of competition and cooperation on motor performance

**DOI:** 10.1371/journal.pone.0237607

**Published:** 2020-09-18

**Authors:** Florian Müller, Rouwen Cañal-Bruland

**Affiliations:** Department for the Psychology of Human Movement and Sport, Institute of Sport Science, Friedrich Schiller University, Jena, Germany; Nanjing University, CHINA

## Abstract

Established research has documented the pervasive influence of incentives (i.e., food, sex, money) on animal and human behavior. Additionally, motivational theories postulating intra–individually stable preferences for specific types of incentives (i.e., motives) highlight that effects of a given incentive are highly dependent on the motive disposition of the individual. Indeed, also research on motor performance has documented the interactive effects of motives and motive–specific incentives on motor outcomes. However, the majority of this research has relied on correlational designs focusing on the effects of the achievement motive, with few studies addressing the role of the affiliation and power motive. In order to extend findings in this domain, we tested whether a fit between individuals’ power (affiliation) motive and incentives of competition (cooperation) would improve motor performance. Following baseline measures, participants performed a dart–throwing task as part of a dyadic performance (i.e., cooperative) or a one–on–one competition scenario. In the dyadic performance scenario, a stronger affiliation motive did not translate to better performance. However, in the one–on–one competition scenario a stronger power motive was associated with better performance. Results highlight the role of the power motive in predicting motor performance, particularly in competitive situations.

## Introduction

Everybody knows that individuals differ substantially in their behavior—both in the choice of behavior they exhibit in the first place, as well as their persistence or success at a given task. For example, people differ in their career choices as well as in their persistence and success at a chosen career [1, 2]. Likewise, individuals show different levels of persistence and success at challenging tasks [3]. Not surprisingly then, such differences have also been documented in early [e.g., 4] and contemporary research [e.g., 5–7] on motor performance [see 8, 9, for an overview].

### Incentives as behavioral determinants

Research on human motivation seeks to explain such differences in the direction (i.e., choosing a behavioral option) and energization (i.e., persistence) of behavior [10, 11]. Early theorizing in the behaviorist tradition [e.g., 12] as well as more recent theories such as expectancy–value approaches [13] have highlighted the role of incentives in shaping behavior. For example, Thorndike’s [14, p. 166] seminal formulation of the “law of effect” posits that behaviors associated with pleasurable consequences (i.e., incentives) will be engaged in more frequently. In contrast, behaviors associated with unpleasant consequences will be engaged in less frequently. This notion has been a cornerstone of theorizing on the mechanisms of motor learning specifically [15, p. 113] and is in general apparent in the common practice of using incentives to guide behavior or improve performance (e.g., verbal encouragement to boost student engagement, [16]; monetary incentives to increase performance of study participants, [17–19]). In addition, the role of incentives in behavioral regulation has more recently been bolstered by linking variations in incentive value to systematic changes in neural correlates, [e.g., 20–22], especially in the context of motor performance [23, p. 219].

Consequently, research on motor behavior has scrutinized the impact of these incentives on motor learning and performance. For instance, performance benefits have been documented, if learning a novel motor task was achieved by rewarding correct responses (i.e., monetary incentive) compared to punishment of incorrect responses [24, 25]. In addition, differential effects of reward and punishment (e.g., awarding vs. deducting monetary incentives) have been shown for motor learning in contrast to retention [26]. Going beyond the domain of such relatively constrained laboratory settings, increased cycling performance has been reported in the presence of competitive incentives [27, see also 28].

On the one hand, these findings attest to the role of incentives in shaping differences in motor learning and performance. On the other hand, both animal [29, 30] and neuroscience research [31–34] as well as research on motivation [35] have argued that an incentive’s properties are inexorably linked to the current needs of the individual, as put succinctly by Lewin (1935): “the dynamics of environmental influences can be investigated only simultaneously with the determination of individual differences” [35, p.73].

### Psychological needs and incentive value

One branch of personality and motivation psychology that has explicitly addressed the issue of individual differences in sensitivity for specific types of incentives is motive research (i.e., Motive Disposition Theory, MDT). Note that the current work’s focus on Motive Disposition Theory is not meant to imply a superiority of this approach compared to the wealth of other theorizing on the role of incentives on behavior. For instance, the role of basic need satisfaction for performance and well–being has been a cornerstone of Self–Determination Theory [36]. Similarly, the effects of both monetary and social incentives have been a staple of research on performance enhancement and behavior change [e.g., 37–39], as well as effort exertion [40].

At the core of MDT though, lies the assumption that individuals are characterized by stable, trait like differences in their preferences for specific stimuli or situations they find rewarding [41; see 42, for an overview]. In short, individual differences in three main motives are distinguished: a high achievement motive endows the “autonomous mastery of challenging tasks” with incentive value. In contrast, for individuals with a high affiliation motive “establishing, maintaining, and restoring positive relationships” and for those with a high power motive “having, physical, mental, or emotional impact on others” constitute relevant incentives [43, p. 603–606]. In line with a person × situation interactionist approach [see 35, p. 73], a given motive will be aroused only in interaction with the specific incentives inherent in a given situation. To give an example, a high power motive should only yield increases in motivation in situations furnished with relevant power incentives (e.g., being able to establish one’s position in a dominance hierarchy, controlling others).

The dependence of stimuli’s incentive value on individuals’ motives has been supported by a number of studies. For instance, Fodor, Wick and Hartsen [44] assessed participants’ power motive and subsequently confronted them with a video of an ostensive job applicant, portrayed either as highly dominant or moderately submissive. The more pronounced participants’ power motive, the stronger their corrugator supercilli (“frown muscle”) activity in response to the dominant applicant; for similar findings see [45–48].

### Motives and motor performance

Beyond these effects on incentives, motives’ impact on motivation and behavior in general (e.g., career success: [2, 49]; persuasion: [50]; see [43, 51, 52] for an overview) and on indicators of motor performance specifically, have been the topic of established research [for a review, see 53].

However, as recently pointed out by Müller & Cañal-Bruland [53], the number of studies addressing the influence of motives on motor performance is relatively low (only 42 publications on the topic have been published in the past 54 years). In addition, the field is characterized by considerable heterogeneity in motive measurement, study design, and outcome variables. For example, the majority of studies has employed correlational approaches and focused on the role of the achievement motive (at the expense of other motives; [e.g., 54–56]). However, such approaches are limited by design, as they are susceptible to confounding influences and do not allow for causal conclusions. Additionally, predominantly focusing on the effects of a single motive (i.e., achievement) entails a neglect of other motives. This constrains theory development as MDT aims to explain and predict effects of all three motives (i.e., achievement, affiliation, and power).

Having said this, a notable exception to this critique is Sorrentino and Sheppard’s [57] experimental study comparing swimmers’ performance when taking part in an individual competition with the very same swimmers’ performance when contributing to a joint team outcome (order counterbalanced). Attesting to the influence of motives on motor performance, the achievement and affiliation motives assessed prior to swimming allowed to differentially predict swimmers’ performance in the two experimental conditions: Swimmers with a strong affiliation motive excelled in the group condition (in contrast to the individual competition condition), whereas the reverse was found for swimmers with a weak affiliation motive. On the one hand, this study is commendable for implementing an experimental approach manipulating the motive specific incentives characterizing the task. On the other hand, the study focused on effects of the affiliation and achievement motive only, whereas no data on the power motive were collected. Even though a handful of conceptually similar studies exist, they either study the effects of a single motive only (i.e., affiliation: [58, 59]; power: [60]) or focus on implicit learning effects [60–62] as opposed to motives’ effects on performance.

Due to these characteristics of the existing literature it was our goal to conceptually build on the findings of Sorrentino and Sheppard [57] by adopting a similar experimental manipulation of motive specific incentives and extending the scope of research to motor performance in a task with an emphasis on coordination [cf. 63], i.e. dart throwing. At the same time, we aimed to expand the scope of previous research by assessing the role of all three motives in the prediction of motor performance.

To this end, participants in the current study performed a dart throwing task in which—following baseline measures—they were either subjected to an individual competition condition or a condition in which they contributed with their performance to a joint (i.e., dyadic) team outcome. More specifically, completing a first block of dart throws served to establish a measure of participants’ baseline dart performance. In a second block of dart throws motive specific incentives (affiliation, power) were manipulated between subjects by having participants perform in either an individual competition condition (power incentive) or team performance condition (affiliation incentive). Consequently, increased performance was expected when individuals’ motives aligned with the motive specific incentives of the task: Participants’ power motive was expected to be especially related to performance in the individual condition. Participants’ affiliation motive was expected to be especially related to performance in the team condition. Finally, even though the experimental manipulations were not targeted at manipulating incentives for the achievement motive directly, the experimental context shares a number of features known to function as achievement incentives (e.g., competing with a standard of excellence, feedback about one’s own performance; see [43]). Because the majority of established studies on the achievement motive has documented positive effects on performance [e.g., 64–67; see 53, for an overview], we also expected a positive influence of participants’ achievement motive on performance (regardless of experimental condition).

## Materials and methods

### Sample

First, a power analysis was run in order to estimate the necessary minimum sample size. We built on Sorrentino and Shepard’s [57] interaction effect of affiliation motivation and experimental condition (*F*-value) as an indicator of target effect size. This value was then converted to a *t*–value ($t=\sqrt{F}$) in order to yield the respective Cohen’s *d* ($d=t\times \sqrt{1/N}=.44$). Targeting a power of at least.8 at an *α*–level of.05 suggested a sample size of *N* = 34. However, because the current research varied situational incentives between subjects (vs. within subjects as in Sorrentino and Sheppard [57]), sample size was doubled to compensate for the reduction in power, resulting in a total of *N* = 68 participants. Those were recruited on campus of the Friedrich Schiller University of Jena. In order to eliminate the chance of interaction effects between participant, ostensible partner, and experimenter sex, an all male sample was recruited. Note that a small number of participants did not follow instructions, e.g., they did throw more than the required number of darts or failed to correctly communicate achieved points to the experimenter. Removal of these participants resulted in a final sample of *N* = 63 (all male, Age: *M* = 23.87, *SD* = 3.02, Range = 20–36). In exchange for participation gift cards were raffled among participants (regardless of actual performance, as detailed below). The study was approved by the Ethics board of the Faculty of Social and Behavioural Sciences at the Friedrich Schiller University of Jena.

### Materials & procedure

#### Motive assessment

Participants completed a version of the Multi-Motive-Grid [68] developed as a semi-projective measure of motives [69, 70] that has been used widely in established research [71–78]. Participants were shown 14 line drawings depicting various social situations (e.g., couples dancing, badminton match) and were asked to indicate for a set of statements whether or not these applied to the depicted situation. The MMG yields two scores for each motive—an approach and an avoidance score (Range: 0–12), with higher values indicating higher motive strength. By subtracting the avoidance from the approach score, one index representing each motive was computed (Range: -12–+12). (Note that due to an error in item assignment to MMG pictures one item of the fear of rejection subscale was not available for a subset of *N* = 47 participants. This resulted in a maximum possible fear of rejection score of 11 for these participants. For these participants the fear of rejection score was transformed such that a score of 11 matched the endpoint of a 0 to 12 scale (new score = old score $\times \frac{12}{11}$). However, using either original or transformed data yielded identical statistical findings.) Even though specific hypotheses were put forward for the power and affiliation motive only, we assessed and report findings on the achievement motive also, in order to provide full information on the impact of all three motives (as argued for in [53], p. 10).

#### Darts: Baseline block

Participants first received instructions on how to perform dart throws by watching a 90 second video of a professional darts player explaining the basics of the task, such as the correct stance and throwing technique (video available at [79]). They then received a set of six metal tipped darts and took position at a distance of 237 cm from the dartboard (indicated by a line on the floor). The target area consisted of a set of 10 concentric circles printed on a sheet of A4 paper, with an increasing number of points (1–10) corresponding to each consecutively smaller circle (diameter of outermost circle: 158 mm). The bull’s eye was positioned at a height of 173 cm above floor level (throwing distance and target position in accordance with official rules of the World Darts Federation [80]). Participants then commenced to take 2 × 6 warmup throws at the dartboard (not scored), followed by 10 sets of 6 throws each, thus yielding a total of 60 baseline throws. After each set of 6 throws, participants removed all darts from the target area and called out the number of points scored with each dart. The experimenter simultaneously entered these points in a data mask at the experimenter’s laptop. After 5 sets of 6 throws, the target sheet was replaced and marked with subject and trial information for later analyses of the exact coordinates for each throw. This baseline assessment of participants’ dart performance served two purposes. First, it allowed to control for differences in participants’ individual performance level in subsequent analyses. Second, it was used to arrive at a reasonable estimate for setting the performance goal in the second block of the dart throwing task based on a participant’s individual performance.

#### Darts: Manipulation block

This assessment of baseline performance was followed by a second block of 10 × 6 = 60 throws. However, before continuing, participants received varying instructions in order to highlight different motive specific incentives of the dart throwing task representing the between-subject factor Incentive (power, affiliation). In the power condition (*N* = 32) participants learned that they had been randomly paired with another participant who had already finished the experiment. Beating this other participant would require them to increase their performance by at least 19 points (pilot work indicated that a gain of 19 points was a reasonable expectation) and determined whether they would enter a raffle of gift certificates among participants. In contrast, participants in the affiliation condition (*N* = 31) learned that they had been paired with another participant who had previously taken part in the study to form a dyadic team. Again, they were told that a performance increase of at least 19 points was needed for the dyadic team to enter a raffle among teams (similar to the relay in [57]). Mirroring the procedure in the baseline block, participants then performed another 60 dart throws.

#### Demographics and comments

Participants completed a short questionnaire assessing both demographic data (age, sex), previous darts experience, whether they noticed anything unusual about the experiment, and their assumptions about the goals of the experiment.

### Procedure

Upon arrival at the laboratory participants were informed about the nature of the upcoming study and gave their informed consent. Specifically, they learned that they were to complete two tasks: After completing a survey on the perception of different pictures (i.e., the Multi-Motive-Grid assessing participants’ implicit motives) they were to take part in a dart throwing task (i.e., assessing participants’ motor performance). Participants were encouraged to contact the experimenters in case they encountered any issues or had further questions. Participants then commenced with the motive assessment phase that was followed by the assessment of baseline performance in darts. This was followed by participants completing the second block where motive specific incentives (power, affiliation) were manipulated between participants. The experiment concluded with the assessment of demographic information and participants’ comments.

### Data analysis

Participants’ actual dart performance was operationalized via their consistency (i.e., variable error) because this measure is most sensitive to changes during practice [see 9, p. 27]. To compute the variable error for each participant and experimental block, locating the position of each impact point on each participant’s four target sheets was implemented as follows: All target sheets were scanned and then presented to coders via a purpose built website. After selecting a target sheet to code, coders commenced to mark a) the center of the target and b) all impact points by highlighting them with the mouse, thus creating a list of xy-coordinates representing all marked points. Based on these data, participants’ variable error was computed and transformed to millimeters. Then, in order to assess the overall influence of experimental conditions on participants’ variable error from baseline to manipulation blocks, a mixed ANOVA was conducted, incorporating the within-subject factor Block (baseline, manipulation), and the between–subject factor Incentive (Power, Affiliation).

Most importantly, in order to assess the influence of participants’ implicit motives in interaction with the experimental condition (power vs. affiliation incentives) on their performance in darts, participants’ variable error scores (lower = better) were subjected to moderated regressions. Specifically, for each motive participants’ variable error was predicted by the respective motive score (z–standardized), the effect coded experimental condition (-1: Affiliation Incentive, +1: Power Incentive) as well as their interaction. Given the directional nature of our hypothesis—the power motive was expected to predict performance in the individual condition, but less so in the team condition (and vice versa for the affiliation motive)—one–sided hypothesis tests were employed for a) the tests for the difference in slopes between conditions and b) for the tests of slopes within the respective conditions. To control for differences in individuals’ baseline performance, variable errors in each experimental condition were first residualized by participants’ variable error in the baseline block. Finally, concerning participants’ guesses about hypotheses, it is important to note that none of them guessed the specific hypotheses tested in the current study and hence the full sample was employed in all reported analyses.

## Results

### Motive specific incentives and performance

**Fig 1:**
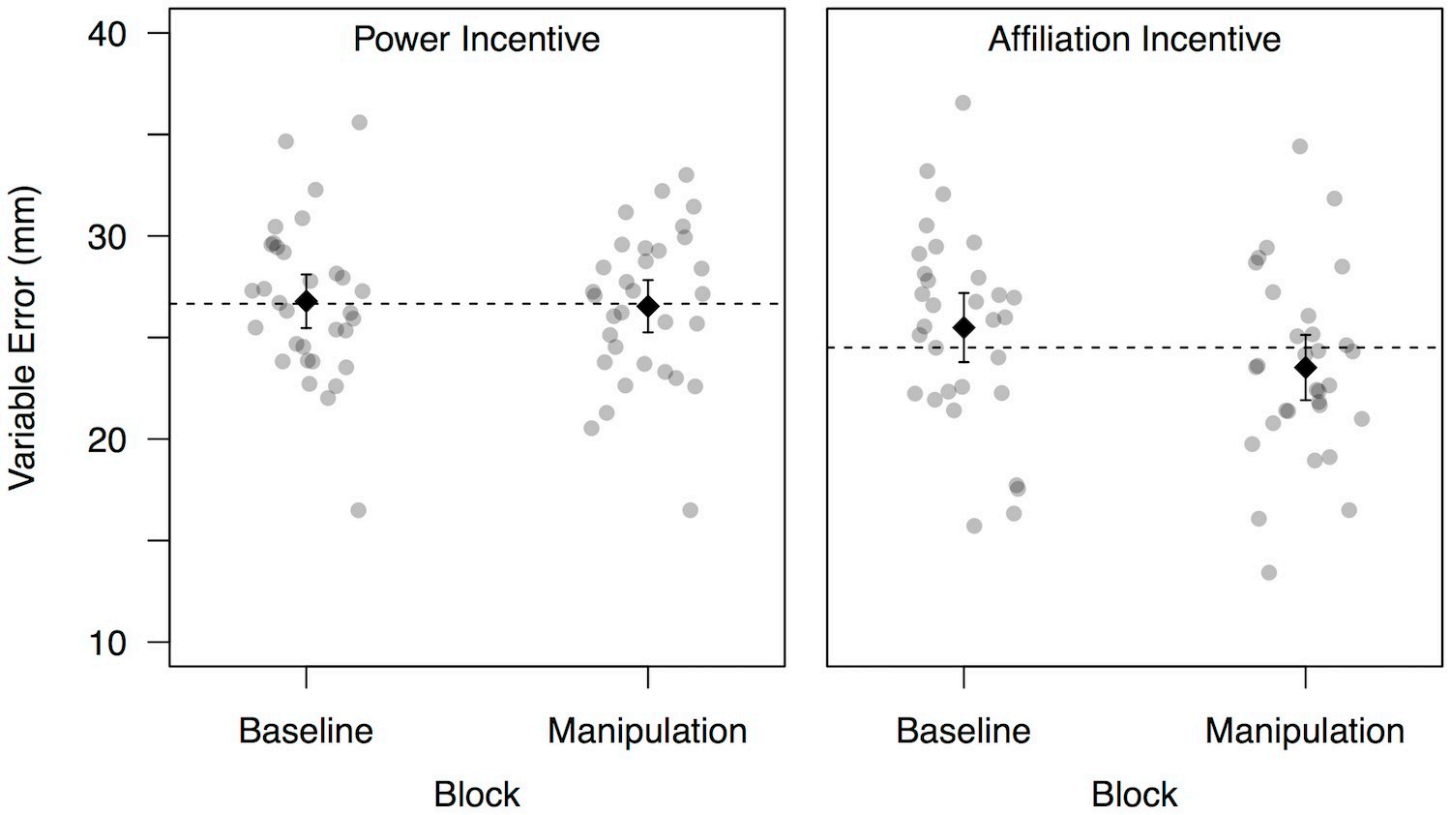
Variable error by block and condition. The influence of experimental block (baseline, experimental) and manipulated incentives (power, affiliation) on participants’ variable error in darts (less error = better performance). Dotted line indicates mean for each incentive, diamonds indicate block means with error bars for 95% CI.

To assess the influence of experimental conditions on participants’ variable error from baseline to manipulation blocks, a mixed ANOVA with the within–subject factor Block (baseline, manipulation), and the between–subject factor Incentive (Power, Affiliation) revealed main effects of Block, *F*(1,61) = 6.14, *p* = .02, $\eta_{p}^{2}=.09$, and Incentive, *F*(1,61) = 4.91, *p* = .03, $\eta_{p}^{2}=.07$. The interaction of Incentive × Block, *F*(1,61) = 3.73, *p* = .06, $\eta_{p}^{2}=.06$, fell short of significance, see Fig 1.

### Motive–incentive congruence and performance

In order to test the influence of participants’ motives in interaction with the motive specific incentives provided by the experimental conditions, participants’ variable error was predicted by each respective motive, the condition, and their interaction using moderated regression analyses.

#### Power motive

**Fig 2:**
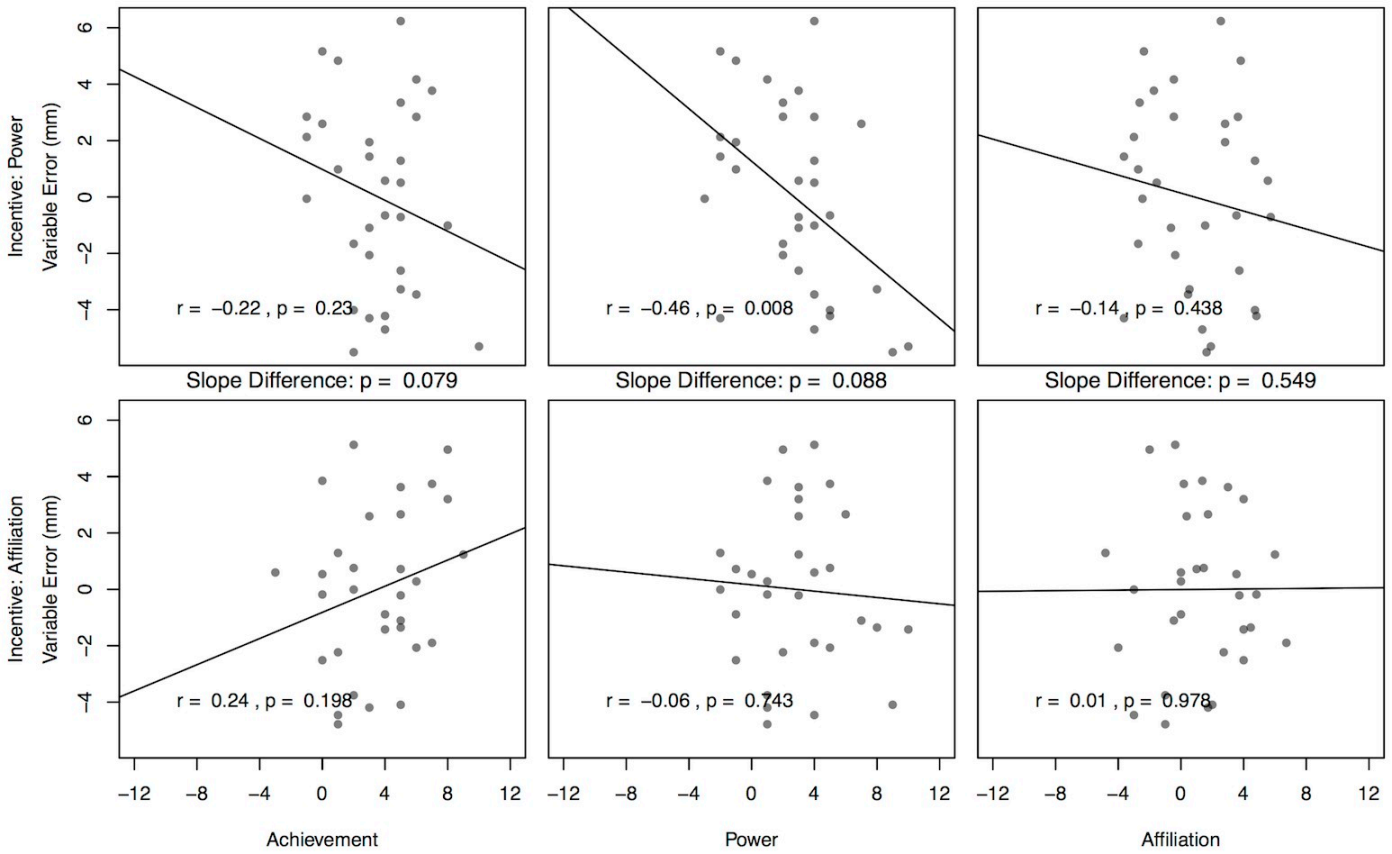
Motives and variable error by condition. Regressions of participants’ variable error (residualized for baseline performance) in the second block (lower = better) on each motive, separately for both experimental conditions (top vs. bottom row). All p-values are two-sided.

As detailed in Table 1, a main effect of the power motive on variable error was qualified by the interaction of power × condition in the hypothesized direction: The relationship between the power motive and variable error was more negative (i.e., higher power = better performance) in the power incentive condition (*p* = .044, one-tailed, see Table 1, Fig 2). Follow up simple regression analyses separately for each condition confirmed a pronounced negative relationship in the power incentive condition, *r* = −.046, *p* = .004 (one–tailed, i.e., a higher power motive yielded reduced variable error). In contrast, this relationship was absent in the affiliation incentive condition, *r* = −0.06, *p* = .743. These relationships are illustrated in Fig 2.

**Table 1:** Moderated regression analyses of participants’ variable error for each of the three motives.

Motive	Predictor	B	SE B	*β*	SE *β*	*t*	*p*
Power	Intercept	0.71	0.50	0.00	0.12	-0.04	.967
	Pow	-0.26	0.12	-0.27	0.12	-2.21	.031
	Condition	0.55	0.50	-0.01	0.12	-0.05	.958
	Pow × Condition	-0.20	0.12	-0.31	0.12	-1.73	.088
	Model: *R*^2^ = .08, *p* = .04						
Affiliation	Intercept	0.07	0.42	0.00	0.13	-0.04	.972
	Aff	-0.08	0.14	-0.07	0.13	-0.57	.574
	Condition	0.07	0.42	-0.01	0.13	-0.03	.974
	Aff × Condition	-0.08	0.14	-0.08	0.13	-0.60	.549
	Model: *R*^2^ = −0.4, *p* = .87						
Achievement	Intercept	0.08	0.63	0.00	0.13	0.02	.988
	Ach	-0.02	0.14	-0.02	0.13	-0.15	.882
	Condition	0.90	0.63	0.00	0.13	0.00	.999
	Ach × Condition	-0.25	0.14	-0.23	0.13	-1.79	.079
	Model: *R*^2^ = .003, *p* = .37						

Note. Reported Model *R*^2^ is adjusted for number of predictors. For purpose of clarity all *p*-values are reported two–sided, even though directional hypotheses were put forward for the interaction effects of power × condition, and affiliation × condition.

#### Affiliation motive

Contrary to expectations no relationships between participants’ affiliation motive and their variable error score were present (see Table 1 and Fig 2).

#### Achievement motive

Similar to the affiliation motive, no effects of participants’ achievement motive on their variable error score were present (see also Table 1 and Fig 2).

### Supplementary analysis on accumulated points

It is of course possible to analyze the effects of participants’ motives in interaction with the experimental condition on actually accumulated points also. Note though, that this approach represents a substantially less fine–grained measure of performance because all hits within the diameter of a specific target ring are assigned the very same performance score (regardless of their exact location on its circumference) thus greatly reducing spatial resolution. For purposes of clarity (we thank an anonymous reviewer for highlighting the benefits of this additional analysis) we nevertheless repeated the previous regression analyses for participants’ accumulated points in the experimental block (after controlling for baseline performance). For participants’ achievement and power motive only a positive main effect of the power motive emerged (*p* = .04, all other *p*’s >.2). In contrast, the affiliation motive’s relationship to accumulated points differed—as hypothesized—depending on condition (*p* = .03, one-tailed). Post-hoc simple regression analyses revealed a negative trend for the relationship of the affiliation motive to performance in the power incentive condition (*r* = −.30, *p* = .10). In contrast this relationship showed the opposite trend in the affiliation incentive condition (*r* = .18, *p* = 0.33). However, we want to emphasize that these findings should be treated very cautiously, due to the low spatial resolution of the dependent variable.

## Discussion

The current work applied motive disposition theory’s classic person × situation interactionist approach positing that motives exert their motivational effects if—and only if—aroused by appropriate incentives [35, p. 73; 41, 62]. We aimed to conceptually replicate and extend previous findings by Sorrentino and Sheppard [57] on the interactive effects of motives and situation characteristics (e.g., motive specific incentives) on motor performance by a) assessing the role of all three motives in motor performance, b) experimentally varying both affiliation and power incentives, and c) assessing the impact of motives on motor performance emphasizing coordination requirements. Results indicate that a more pronounced power motive was associated with improved performance if—and only if—the experimental condition provided power incentives (i.e., the individual competition condition). In contrast, no effects emerged for the relationship of the affiliation motive and performance, as well as for the achievement motive and performance. (Note that there was also a trend for the hypothesized pattern concerning the moderation of the affiliation motive’s relationship to participants’ accumulated points. Due to the shortcomings of this measure compared to indicators of performance consistency, we urge the reader to treat this latter finding cautiously).

### Relationship to previous findings

Most importantly, in three aspects the present study goes beyond previous findings. First, by focusing on the role of the power and affiliation motive we draw attention to two motives underrepresented in established motive research. This is underlined by the fact that 76% of the published studies reviewed by Müller and Cañal-Bruland [53] assessed the role of the achievement motive only. Second, the current study utilized an experimental manipulation of motive specific incentives that goes beyond the majority of correlational approaches in the field [see 53, p. 10]. Finally, to the best of our knowledge, the current study is the first that documents the effects of the power motive on motor performance.

This is due to the fact that those experimental studies explicitly addressing the role of the power motive have focused on the relationship to (implicit) motor learning such as sequence learning. Motor learning is generally defined as “a change in the capability of a person to perform a skill […] inferred from a relatively permanent improvement in performance as a result of practice or experience” [81, p. 257]. Studies on the relationship between the power motive and implicit motor learning used similar tasks (e.g., connecting numbers in a matrix in ascending order) as an index of implicit learning and manipulated the presence of power motive incentives by either manipulating whether participants won or lost [60, 62] or by pairing sequence learning with pictures of power motive congruent (e.g., submissive) or incongruent (e.g., dominant) faces [61]. In general, results from these studies indicate increased implicit learning in the case of motive–incentive congruence.

The current study complements these findings by additional evidence about the role of the power motive not only for motor learning, but for motor performance generally defined as “the behavioral act of executing a specific skill at a specific time and in a specific situation” [81, p. 257]. Findings concerning the affiliation motive however, were in contrast to previous research indicating that individuals high in affiliation show better performance in a team condition [57]. Readers may recall that there was indeed a trend for the hypothesized relationship of the affiliation motive if participants’ accumulated points were used as performance indicator. However, as discussed previously (see p. 7), these results should be treated with caution and await further research.

One reason for these divergent findings might be the use of different motive measures. For instance, Sorrentino and Sheppard employed an aggregate of different motive measures, that is, a difference score of the projective TAT based affiliation motive [82, Appendix III] and the self–reported fear of rejection score (Interpersonal Opinion Questionnaire, [83]). In contrast, in employing the Multi-Motive-Grid [68] the current study assessed all three motives with a single, unified measure. We want to point out that even though the MMG has enjoyed widespread use in established research (see Methods section for an elaboration), there has also been debate concerning its validity as a measure of implicit (vs. explicit) motives [see 84, p. 79]. Acknowledging these issues, we caution against interpreting our findings as evidence for the impact of implicit motives specifically. Clearly, future research is needed to address the specific role of implicit and explicit motives in motor performance, as we have argued elsewhere [53].

Nevertheless, the fact that relationships of the affiliation motive to motor performance have been documented in other studies employing a variety of motive measures [e.g., 58, 59] suggests that the absence of affiliation motive effects might not be solely due to differences in motive assessment.

### Motive arousal: A question of appropriate incentives

Specifically, this may have also been caused by a failure to sufficiently arouse the affiliation motive in the first place. For instance, [57] as well as [58] employed the experience of actual contact with their teammates as an incentive for the affiliation motive. In order to increase experimental control of external variables, the current study opted for an alternative approach, namely telling participants that they were part of a two-person team (similar to [59]). Therefore, we cannot rule out that the levels of motive arousal induced with our method might be inferior (and perhaps even insufficient) to alternatives entailing actual contact with others.

In addition, the current data did not show evidence for a relationship of the achievement motive to participants’ performance. At first, this finding seems at odds with the bulk of the literature documenting such an association [see 53]. However, in contrast to the current paradigm, the overwhelming majority of these studies did not introduce incentives that directly targeted motives other than the achievement motive. In fact, to the best of our knowledge, the only instance of such a design is Kelly, Rawson, and Terry’s [85] research on the moderating effects of competitive vs. cooperative task settings on the relation between the achievement motive and performance: Participants high in achievement were most successful in the cooperative setting, whereas those low in achievement excelled in the competitive setting. It is noteworthy, that effects of such rival incentives, that is, of incentives for one specific motive (e.g., dominance incentive for power) on a second, unrelated motive (e.g., achievement) have been largely neglected by research (with the exception of research on motive–goal congruence addressing conceptually related issues [e.g., 86–89]). Consequently, it may be fruitful to consider these aspects in future research.

Finally, the current findings were documented in a sample of undergraduate university students at a sports science institute. Given the fact that motive by situation interaction effects have been reported in the literature for a wide variety of samples [see 53, for a review] we expect our findings to generalize to other samples also. Note though, that only a small subset of participants had considerable practice in darts (i.e., only 12% of participants played more than once a month). Given that changes in variable error may be less likely in highly skilled participants results may be less pronounced in samples with a higher proportion of experienced players.

To sum up, the current work addresses an important gap in the literature. On the one hand, we provide first evidence on the influence of the power motive on motor performance in competitive performance environments—a prime example of ecological valid behavior [90]. Our findings augment and extend previous knowledge on the effects of motives on motor performance by going beyond the influence of the achievement motive only and incorporating the measurement of all three motives instead. Given the inconsistent findings on the role of the affiliation motive in motor performance to date, future research is needed to further unravel the mechanisms that drive motives’ effect on motor performance [see 53].

## Supporting information

S1 Data (CSV)
